# The mediating role of depression and moderating role of self-consciousness in the relationship between parental negative control and adolescents’ perceived school climate

**DOI:** 10.1186/s13034-025-00896-3

**Published:** 2025-04-02

**Authors:** Jiajie He, He Xiao, Jian Mao, Peizhi Zhong, Junfeng Wei, Wenhao Gu, Yangang Nie

**Affiliations:** 1 Research Center of Adolescent Psychology and Behavior, School of Education, Guangzhou University, Guangzhou Higher Education Mega Center, 230 Waihuan Rd West, Panyu District, Guangzhou, Guangdong China; 2 Center for Studies of Psychological Application, South China Normal University, Guangzhou, China

**Keywords:** Parental negative control, Depression, Perceived school climate, Self-consciousness, Adolescent

## Abstract

**Background:**

A positive school climate benefits adolescents in multiple developmental dimensions. However, theoretical frameworks suggest that detrimental parenting practices can trigger adolescents’ negative emotional states, which may skew their perceptions of school climate. Although self-consciousness, a critical intrapersonal trait, may mitigate these adverse effects, limited research has empirically explored its moderating role in the context where negative parenting influences adolescents’ mental health and school-related outcomes. Thereby, this study investigates how parental negative control influences adolescents’ perceived school climate via depression, with self-consciousness moderating the link between parental negative control and depression.

**Methods:**

A short-term longitudinal design was employed, with the data collected from a sample of Chinese adolescents (N_T1_ = 733, N_T2_=711). Self-reports were used to measure parental negative control, depressive symptoms, self-consciousness, and perceived school climate. Structural equation modeling was conducted to assess the mediating effect of depression and the moderating effect of self-consciousness.

**Results:**

The findings show that depression mediates the relationship between parental negative control and perceived school climate. Adolescents experiencing higher levels of negative control reported more depressive symptoms, which were associated with lower perceptions of school climate. Self-consciousness moderates the link between parental negative control and depression, with adolescents exhibiting higher self-consciousness showing less vulnerability to the adverse effects of negative control.

**Conclusions:**

This study reveals the detrimental impact of parental negative control and depression on adolescents’ perceived school climate while highlighting the protective role of self-consciousness. Interventions can target families engaged in maladaptive parenting practices and adolescents with psychopathological symptoms, focusing on enhancing family dynamics and adolescents’ self-processes.

## Background

A positive school climate is linked with enhanced academic motivation [1, 2], an improved sense of belonging, reduced behavioral issues, and better psychological well-being among students [3, 4]. Conversely, students with a negative perception of school climate are more likely to report diminished learning motivation, decreased interpersonal engagement, and heightened psychological distress [4, 5]. Adolescence is marked by a tendency to interpret surroundings through a more emotional and less rational lens [6, 7]. This renders adolescents’ schemas, attitudes, and evaluations of external environments subject to their emotional states [8]. As such, negative emotional states may skew adolescents’ view of school including school climate, teacher support, and peer relationship.

Parents inarguably play a profound role in shaping adolescents’ emotional well-being. Detrimental parenting practices, such as parental negative control, have been associated with heightened psychopathology such as internalizing behaviors and externalizing behaviors [9, 10]. Yet, adolescents are not merely passive recipients of environmental influences, they actively contribute to their development through interactions between their individual characteristics and social contexts, which jointly influence health outcomes. Despite the prior studies investigating the moderation role of self-processes, such as self-regulation [11, 12] and self-compassion [13], with regard to the effects of parenting on adolescent health, limited attention has been paid to self-consciousness. This multi-faceted concept, which involves many critical intrapersonal attributes including self-evaluation [14, 15], may interact with parental control to affect adolescents’ emotional well-being and school-related outcomes. Adolescents with higher levels of self-consciousness may be less vulnerable to adverse effects of negative external influences [16] such as negative parenting styles [17] and deviant peer relationship [18, 19]. In contrast, those with lower levels of self-consciousness may internalize adverse experiences, which intensifies their downstream effects. Besides, in the extant body of research on psychopathological symptoms, such as depression or anxiety, these mental health problems are often the endpoint in research design, their extended impacts within explicit social contexts are not examined. This omission constrains our contextualized understanding of the interplay between adolescents’ emotional and cognitive processes in influencing their interpretation of external realities.

To further understand how negative parenting practices and adolescent vulnerabilities converge to affect their perceptions and well-being within educational contexts, this study aims to assess a moderated mediation model in which parental negative control affects adolescents’ perceived school climate indirectly through depression, with self-consciousness moderating the relationship between negative control and depression.

### The impact of parental negative control on adolescents’ depressive symptoms: an ecological system perspective

Bronfenbrenner’s ecological systems theory [20] has been extensively used as a robust framework for understanding how parenting behaviors, situated in the proximal microsystems, significantly influence adolescent development, for better or worse. Within this framework, prior research has consistently established a correlation between harmful parenting practices (e.g., harsh parenting, parental phubbing, and parental psychological control) and depressive symptoms in adolescents [21, 22], with underlying mechanisms including experiential avoidance, negative self-cognition, rumination, victimization, parental academic pressure, and the satisfaction of individuals’ basic psychological needs [10, 23].

The current study targets parental negative control, a type of parenting practice characterized by manipulative behaviors such as guilt induction, invalidation of autonomy, and excessive criticism [24, 25]. These parental behaviors are particularly prevalent in China probably because of the cultural norms that grant parents a sense of authority to justify holding sway over their children under the guise of love. However, parents often fail to recognize such behaviors as controlling while overlooking the necessity and significance of setting healthy boundaries [26, 27]. Empirical evidence has repeatedly linked these controlling parenting styles to increased depressive symptoms in adolescents [28, 29]. Soenens et al. [30] further contend the adverse impacts of psychological control on depressive symptoms apply across cultural contexts.

### The effect of depression on perceived school climate—an affective-cognitive perspective

While prior theories (e.g., Beck’s cognitive theory) [31, 32] deem those negative cognitive structures, which result from personal histories, genetic factors, and social environments, are the antecedents of emotional disturbances, growing evidence suggests a reciprocal relationship. Such that, depressive symptoms not only stem from but also reinforce negative cognitive patterns, leading individuals to fixate on the defective aspects of themselves, the future, and the world. A cognitive-affective-behavioral model proposed by Pachankis [33] emphasizes the interconnectedness of cognition, affection, and behaviors, implying that these components can feed one another and form a dynamic loop to perpetuate psychological dysfunction.

School climate refers to the quality and character of school life as experienced by students, parents, and staff [4, 34]. It encompasses multiple dimensions such as the school’s physical and emotional environment, teacher-student relationship, peer relationship, and the clarity and consistency of school regulations. School climate reflects norms, values, and expectations that support students’ sense of safety and belonging [4, 35]. A positive school climate has been shown to reduce stress and depression [36], enhance life satisfaction, self-efficacy, and academic motivation [2], ultimately contributing to improved mental health and a more optimistic outlook on both personal and academic life [3, 5].

Through the lens of the cognitive-affective-behavioral model [33], adolescents with depressive symptoms may perceive the school climate in a negative and distorted light. The selective attention to negative stimuli and the difficulty of disengaging from negative information, both of which feature depression [37], likely lead to negative emotions and attitudes toward school. In the case where negative school-related events emerge, such as academic setbacks, teachers’ unfair treatment, and conflict with peers, depressed adolescents may fixate on these experiences and struggle to move past them. Over time, this attentional bias not only contributes to the excessive attention to negative aspects of the school but also causes a tendency to interpret neutral or ambiguous experiences in school as unsupportive or hostile.

The literature is replete with evidence illustrating that adolescents’ perceived school climate served as the precursors to mental health [38, 39], particularly depressive symptoms [12, 36], yet, relatively few studies have explored how depression shapes perceptions of school climate. Several studies [40, 41] employed cross-sectional design to examine the association between perceived school climate and depressive symptoms, yielding inconsistent results. Despite the theoretical support for the potential influence of depressive symptoms on perceptions of school climate, this directionality of the relationship remains underexplored.

Research revealed that depressed adolescents tend to report higher levels of academic dissatisfaction, peer rejection, and teacher criticism [42, 43]. While the negative perceptions of themselves and their peers in adolescents with depressive symptoms partially reflected actual social difficulties, the degree of negativity in these perceptions exceeds what was objectively warranted by the situation [44]. This evidence of cognitive distortion in social perceptions among depressed adolescents suggests the possibility of a distorted view of their school environment and experiences.

### Self-consciousness as a moderating factor: a cognitive vulnerability-stress perspective

Previous studies have highlighted the interaction effects of factors such as neuroticism [45], peer acceptance [23], mindfulness [46], and parental warmth [47] on the relationship between parenting and adolescent depression. Beyond these, growing evidence has identified the moderating role of self-processes in mitigating the impacts of harmful parenting practices on adolescent development. For instance, Liu and Hu [13] demonstrated that self-compassion moderates the relationship between harsh parenting and adolescent depression. The research by Wang and Jiang [48] revealed that self-regulation buffers the impacts of parental neglect on cyberbullying perpetration. Similarly, Crespo et al. [11] reported that self-regulation moderates the association between household chaos and child deviant behavior. These findings indicate the adaptive function of self-processes in lessening adolescents’ vulnerability to environmental stressors.

However, limited attention has been given to adolescents’ self-consciousness. In the common discourse, self-consciousness refers to a heightened awareness of oneself as an individual, distinct from the environment and others, akin to self-awareness [49, 50]. However, in the current study, self-consciousness is conceptualized more closely with self-concept, encompassing a set of beliefs and perceptions that individuals hold about themselves as well as the associated self-processes including self-evaluation and self-control [14, 51]. Research has documented that self-consciousness is fundamental to emotional and cognitive development in adolescents [52, 53]. A high level of self-consciousness can serve as a protective factor, enabling adolescents to critically evaluate external stressors and diminish their negative effects [54].

The cognitive vulnerability-stress model [55] offers a compelling framework for examining the interplay between self-consciousness, parenting practices, and depressive symptoms. This model proposes that cognitive vulnerabilities, such as negative inferential style, can interact with negative stressful events to increase the risk of depression. In line, the hopelessness theory of depression [56] posits that individuals with a negative inferential style are inclined to interpret adverse events as persisting and pervasive, catastrophize their consequences, and attribute them to personal deficiencies [57, 58]. Empirical studies have identified that cognitive vulnerabilities can be activated by schema-consistent stressful events, heightening the likelihood of developing depressive symptoms [59, 60]. In this context, adolescents with low self-consciousness likely exhibit cognitive vulnerabilities, such as distorted self-evaluation and a diminished self-image [61]. Parental negative control, representing an environmental stressor, can exacerbate these vulnerabilities. As such, adolescents with low self-evaluation may internalize negative feedback from parents, leading to feelings of inadequacy and emotional distress. Conversely, those with stronger self-consciousness may be better equipped to navigate negative parental behaviors, thereby buffering their adverse impacts.

### The current study

**Fig. 1:**
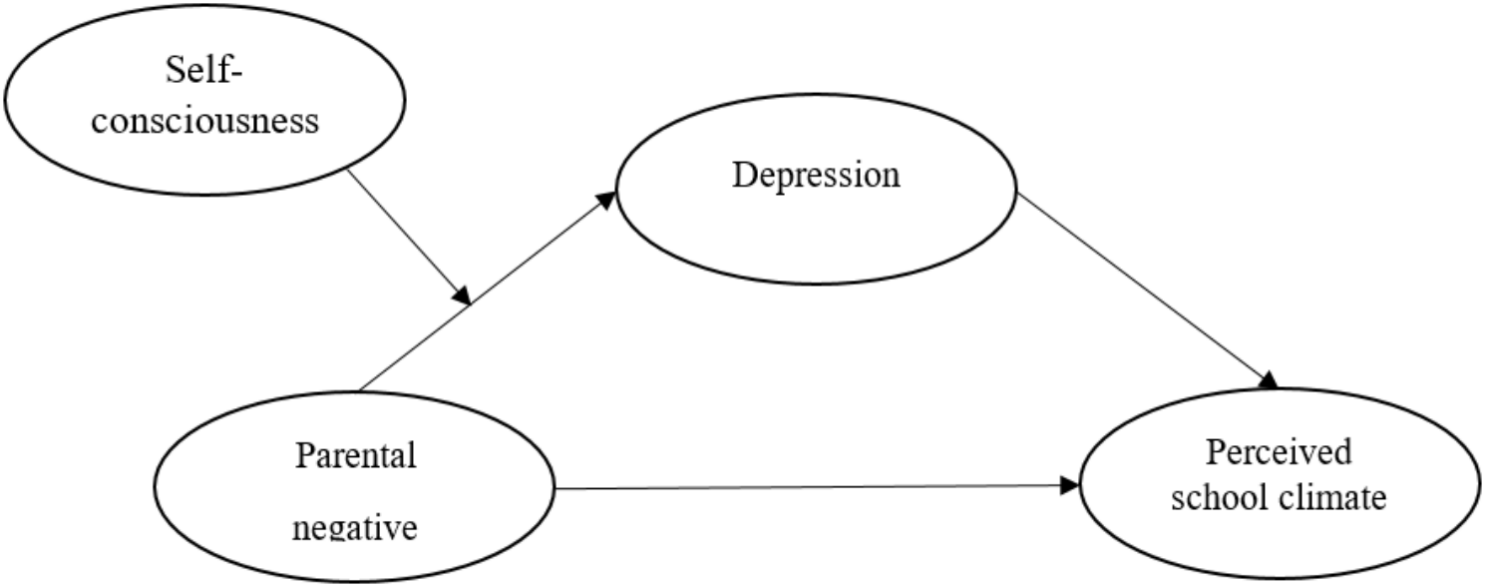
A moderated mediation model of parental negative control, depression, self-consciousness and perceived school climate

Multiple models collectively constitute the theoretical foundation of the current study. Bronfenbrenner’s ecological systems theory provides an overarching framework, guiding the focus on how proximal environmental stressors (e.g., parental negative control) shape adolescent development. Within this broad ecological perspective, the study is anchored in the cognition-related models. Specifically, the cognitive-affective-behavioral model elucidates how depression could lead to maladaptive cognitive patterns that may shape adolescents’ perceptions of the external world (e.g., perceived school climate). The cognitive vulnerability-stress model offers a nuanced perspective on this process by highlighting how specific intrapersonal factors (e.g., high self-consciousness) may serve as cognitive coping strategies that buffer the detrimental effects of stressors. Integrating these cognitive models within the wide ecological systems framework allows for a more nuanced understanding of the mental and cognitive mechanism by which parental negative control impacts adolescents’ perceived school climate. Given this theoretical frameworks and gaps in the literature, the current study constructs a latent variable moderated mediation model (Fig. 1) and proposes the hypotheses as follows: (1) Depression mediates the relationship between parental negative control and perceived school climate; (2) Self-consciousness moderates the relationship between parental negative control and depression, with the relationship being weaker for adolescents with higher levels of self-consciousness compared to those with lower levels.

## Methods

### Procedure

The present study was a component of a large research project aimed at examining the transactional impacts of gene-environment interactions on the development of self-control in adolescents. This study was approved by the Ethics Review Committee (IRB) of the authors’ affiliated institution. Convenience sampling was used to recruit participants from three high schools in a city located in the southern region of China. Only students from the 7th and 10th grades were sampled as they would be able to continue to participate in the follow-up assessments during the next year (8th and 11th grader would enter their final year in the junior and senior high school respectively, and are normally not permitted by school to partake in any forms of research). Prior to signing consent forms, all participants and their parents were made aware of their rights (e.g., freely withdraw from the study at any time without any penalty). The research procedure was conducted in a way strictly abiding by the institution-approved research protocol. Well-trained data collectors visited schools to distribute questionnaires to the students after informing them of the research goal and assessment guidelines.

Students took the surveys in November 2021 (T1) and May 2022 (T2). Although the COVID-19 pandemic had not yet ended during data collection, there were very few cases in the researchers’ city. As a result, students had fully resumed in-person teaching after May 2020. Additionally, between the two waves of data collection, there was only one brief one-week period of online learning, which occurred more than a month before the T2 data collection. Therefore, we believe that the impact of COVID-19 and online teaching on our study was limited.

### Participants

A total of 733 students participated in the data collection at T1. Due to transfers and leaves of absence, T2 participants included 711 students (*Mean*_age_ = 14.30 years, *SD* = 2.26, 48.1% female, 47.3% 7th grade) in the 7th and 10th grades. 70% of participants resided in cities, with the remaining participants distributed across county, town, or country. Nuclear families comprising 88.7% of participants’ family structures. 54.4% of the participants cohabited with at least one parent for five or more days per week. Approximately 69.5% of the participants indicated having one or more siblings. 71.6% of participants rated the family economic status as average Parental education levels ranging from elementary school to graduate degrees.

### Measures

#### Parental negative control (T1)

Parental negative control was assessed by the negative dimension of parental monitoring scale devised by Zhang et al. [25]. The subscale comprised 4 items, assessing the parental negative control and feedback toward adolescents. Adolescents were required to answer on a 5-point scale (1 = *not true at all* to 5 = *absolutely true*). Example items include “If I disappoint my parents, they will ignore me” and “If I don’t do things the way my parents want, they will get very angry”. Higher mean scores indicate higher levels of parental negative control. The scale in the current study demonstrates good reliability and validity (Cronbach’s alpha = 0.784, *χ*^*2*^/df = 1.179, CFI = 1.000, TLI = 0.999, RMSEA = 0.016, 90%CI [0.000, 0.078], SRMR = 0.009).

#### Depression (T2)

Depression was measured by the Center for Epidemiologic Studies Depression Scale [62]. This is a one-dimensional questionnaire consisting of 10 items that assess adolescents’ depression state. Adolescents were required to indicate how often each statement happened to them on a 4-point scale (0 = *never or rarely* to 3 = *almost always*). Example items include “I have trouble keeping my mind on what I was doing” and “I feel very depressed”. Higher mean scores reflect higher levels of depression. The scale in the current study demonstrates good reliability and validity (Cronbach’s alpha = 0.858, *χ*^*2*^/df = 2.722, CFI = 0.985, TLI = 0.972, RMSEA = 0.049, 90%CI [0.035, 0.064], SRMR = 0.023).

#### Self-consciousness (T1)

Self-consciousness was assessed by the Adolescents’ Self-Consciousness Questionnaire [54]. It is an indigenous scale specifically developed to assess various types of self-consciousness among Chinese adolescents, it was also proven to be applicable in other cultural contexts [51, 63]. This questionnaire included 64 items of three dimensions: Self-Evaluation, Self-Experience, and Self-Control. Adolescents were required to answer on a 5-point scale (1 = *not like me at all* to 5 = *like me very much*). Example items include “I am satisfied with my appearance” and “I am good at communicating with people”. Higher mean scores indicating higher self-consciousness. The scale in the current study demonstrates good reliability and validity (Cronbach’s alpha = 0.954, *χ*^*2*^/df = 2.755, CFI = 0.990, TLI = 0.971, RMSEA = 0.050, 90%CI [0.033, 0.066], SRMR = 0.023).

#### Perceived school climate (T1, T2)

Perceived school climate was measured by the Delaware School Climate Surveys-Student (DSCS‐S) [64]. This 28-item questionnaire consists of seven subscales: teacher–student relations, student–student relations, school‐wide student engagement, clarity of expectations, fairness of rules, school safety, and school‐wide bullying. The items are rated on a four‐point scale (1 = *strongly disagree* to 4 = *strongly agree*). Example items include “Students care for each other” and “Teachers care about their students”. Higher mean scores indicate higher perceived school climate. The scale in the current study demonstrates good reliability and validity at both T1 and T2 (T1: Cronbach’s alpha = 0.935, *χ*^*2*^/df = 2.257, CFI = 0.997, TLI = 0.990, RMSEA = 0.042, 90%CI [0.010, 0.072], SRMR = 0.011; T2: Cronbach’s alpha = 0.947, *χ*^*2*^/df = 2.535, CFI = 0.997, TLI = 0.990, RMSEA = 0.046, 90%CI [0.020, 0.074], SRMR = 0.014).

#### Demographic covariates (T1)

Adolescent age, gender (0 = male, 1 = female), school type (0 = junior high school, 1 = senior high school), and socioeconomic status (1 = impoverished, 2 = below average income, 3 = average income, 4 = above average income, 5 = high income) were included as demographic covariates in analyses because their significant associations with perceived school climate in previous studies [65].

### Data analyses

The current study used SPSS 27.0 to examine attrition patterns and common method bias and to obtain descriptive statistics and correlations. Subsequently, Mplus 8.0 was employed to examine both the measurement model and the path models. In the measurement model, confirmatory factor analysis was conducted to process the four key variables—parental negative control, self-consciousness, depression, and perceived school climate—as latent constructs, thereby accounting for measurement error and enhancing the reliability and validity of the analyses. After ensuring that the measurement model demonstrated a good fit to the data, relevant paths were specified to test the mediation model and the moderated mediation model. In constructing the latent variable model, self-consciousness and perceived school climate were modeled based on their respective dimensions, whereas parental negative control and depression, as unidimensional scales, were represented using all original observed indicators. Bootstrap analyses with 5,000 resamples were performed to assess the significance of the effects. When testing the moderation effect of self-consciousness on the relationship between parental negative control and depression, the low level of self-consciousness was defined as one standard deviation below the mean, while the high level was defined as one standard deviation above the mean. Demographic covariates and the initial level of perceived school climate (i.e., at T1), were controlled for as covariates in the models. Following Hoyle’s [66] recommendations, acceptable model fit was evaluated using the following criteria: *χ*^*2*^/df < 5, CFI and TLI > 0.90, RMSEA and SRMR < 0.08.

## Results

### Attrition analysis

We categorized participants into two groups: those who dropped out at T2 (Group1, *n* = 22) and those who had completed both waves of measurement (Group2, *n* = 711). The results indicated that two group did not differ in gender (*χ*^*2*^(1) = 0.060, *p* =.807), school type (*χ*^*2*^ (1) = 3.744, *p* =.053), socioeconomic status (*t* (731) = 0.234, *p* =.815), parental negative control (*t* (21.619) = 1.609, *p* =.132) and T1 perceived school climate (*t* (731) = 1.406, *p* =.160). However, the two groups significantly differed in age (*t* (22.485) = -2.076, *p* =.049) and self-consciousness (*t* (731) = -5.368, *p* <.001), with Group 1 exhibiting lower levels of both age and self-consciousness. Based on Little’s Missing Completely at Random (MCAR) test, the data were determined to be MCAR (*χ*^*2*^ (503) = 548.647, *p* =.078). Therefore, in subsequent analyses, we excluded Group 1 and included only participants who completed both waves of measurement (Group 2). This sample meets the minimum sample size requirements proposed by Kline [67] and Hair et al. [68], and preliminary examination of the measurement model’s fit indices revealed a good model fit (*χ*^*2*^/df = 2.456, *p* <.001, CFI = 0.962, TLI = 0.953, RMSEA = 0.045, 90%CI [0.041, 0.050], SRMR = 0.054, AIC = 32481.971, BIC = 32934.071).

### Common method bias

Given that all questionnaires were self-reported, Harman’s single-factor test was performed to assess the risk of common method bias [69]. The results indicated that the eigenvalues of all 25 factors exceeded 1, with the first factor accounting for only 21.99% of the variance. This suggests the low likelihood of the common method bias in this study.

### Preliminary analyses

Means, standard deviations, and bivariate correlations between the variables are presented in Table 1. As hypothesized, significant correlations were observed among the key variables, with the directionality of these correlations aligning with the expected patterns. Specifically, parental negative control was positively associated with depression, while both of these variables exhibited negative associations with self-consciousness and perceived school climate at T1 and T2. Furthermore, self-consciousness and perceived school climate at both T1 and T2 were positively correlated with one another.

**Table 1:** Bivariate correlation between variables

	M	SD	1	2	3	4	5	6	7	8	9
Demographic covariates											
1. Age	14.30	1.51	1								
2. Gender	0.48	0.50	− 0.06	1							
3. School type	0.53	0.50	0.97***	− 0.47	1						
4. Socioeconomic status	3.11	0.55	− 0.09*	− 0.02	− 0.09*	1					
Main variables											
5. Parental negative control	2.34	0.91	− 0.11**	0.03	− 0.11**	− 0.06	1				
6. Depression	1.03	0.61	0.04	0.23***	0.05	− 0.11**	0.33***	1			
7. Self-consciousness	3.64	0.53	− 0.06	− 0.14***	− 0.05	0.18**	− 0.38***	− 0.53***	1		
8. Perceived school climate (T1)	3.17	0.41	− 0.06	− 0.01	− 0.06	0.10*	− 0.26***	− 0.31***	0.51***	1	
9. Perceived school climate (T2)	3.09	0.45	-12**	0.00	-0.10**	0.17***	− 0.26***	− 0.40***	0.45***	0.60***	1

Gender: 0 = male, 1 = female; School type: 0 = junior high school, 1 = senior high school;

^*^p <.05, ** *p* <.01, *** *p* <.001; T1 = Time 1, T2 = Time 2

### Testing for mediation effect

We then tested our first hypothesis that depression mediated the relationship between parental negative control and perceived school climate. Demographic covariates and baseline perceived school climate were treated as covariates in the analysis. The goodness-of-fit indices demonstrated that the mediation model provided adequate fit to the empirical data (*χ*^*2*^/df = 2.375, *p* <.001, CFI = 0.945, TLI = 0.935, RMSEA = 0.044, 90%CI [0.041, 0.047], SRMR = 0.073, AIC = 39253.607, BIC = 39988.841).

**Fig. 2:**
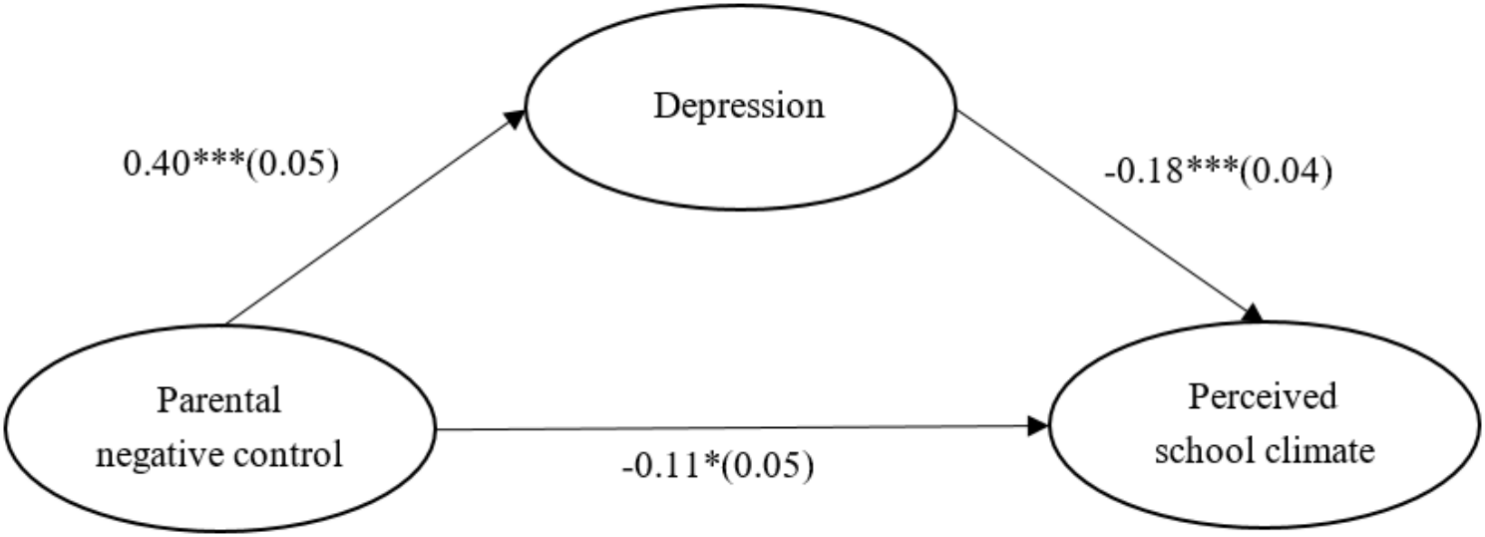
The mediating effect of depression between parental negative control and perceived school climate. Standardized coefficients are reported. * *p* <.05, ** *p* <.01, *** *p* <.001.

The results are presented in Table 2; Fig. 2 which indicate that the path from parental negative control to depression was significant (*β* = 0.40, *SE* = 0.05, *p* <.001, 95% CI [0.302, 0.505]), and the path from depression to perceived school climate was also significant (*β* = -0.18, *SE* = 0.04, *p* <.001, 95% CI [-0.268, -0.096]). After controlling for depression, the path from parental negative control to perceived school climate was significant (*β* = -0.11, *SE* = 0.05, *p* =.026, 95% CI [-0.199, -0.014]). Additionally, bootstrapping analyses indicated that depression significantly mediated the relationship between parental negative control and perceived school climate (indirect effect = -0.07, *SE* = 0.02, *p* =.001, 95% CI = [-0.117, -0.037]. The mediation model explained 49.85% of the variance in T2 perceived school climate.

**Table 2:** The output of the mediation model

	Depression		Perceived school climate (T2)	
	β(SE)	95% CI	β(SE)	95% CI
Main variables				
Parental negative control	0.40*** (0.05)	0.302, 0.505	-0.11* (0.05)	-0.199, -0.014
Depression			-0.18*** (0.04)	-0.268, -0.096
Covariates				
Age	-0.15 (0.13)	-0.413, 0.119	-0.21 (0.12)	-0.443, 0.027
Gender	0.24*** (0.04)	0.164, 0.307	0.04 (0.03)	-0.023, 0.106
School type	0.24 (0.13)	-0.021, 0.496	0.13 (0.12)	-0.105, 0.361
Socioeconomic status	-0.07 (0.04)	-0.139, 0.004	0.09** (0.03)	0.028, 0.148
Perceived school climate (T1)			0.52*** (0.04)	0.453, 0.594
Mediation effect	-0.07*** (0.02)	-0.117, -0.037		

Gender: 0 = male, 1 = female; School type: 0 = junior high school, 1 = senior high school

^*^p <.05, ** *p* <.01, *** *p* <.001; T1 = Time 1, T2 = Time 2

### Testing for moderated mediation effect

As suggested by Maslowsky et al. [70], the moderated mediation model (H0 Value = -19398.794) was compared against the mediation model (H0 Value = -19465.803). The log-likelihood ratio test yielded a difference of *D* = 134.018, significantly exceeding the critical threshold for statistical significance (*χ² (1)* = 134.018, *p* <.001). This shows that the moderated mediation model offers a significantly better representation of the data. Therefore, the moderated mediation model was further used to test second hypothesis on whether self-consciousness would moderate the association between parental negative control and depression. The goodness-of-fit indices for this model (AIC = 39123.588, BIC = 39867.956) indicated an adequate fit to the empirical data^1^.

**Fig. 3:**
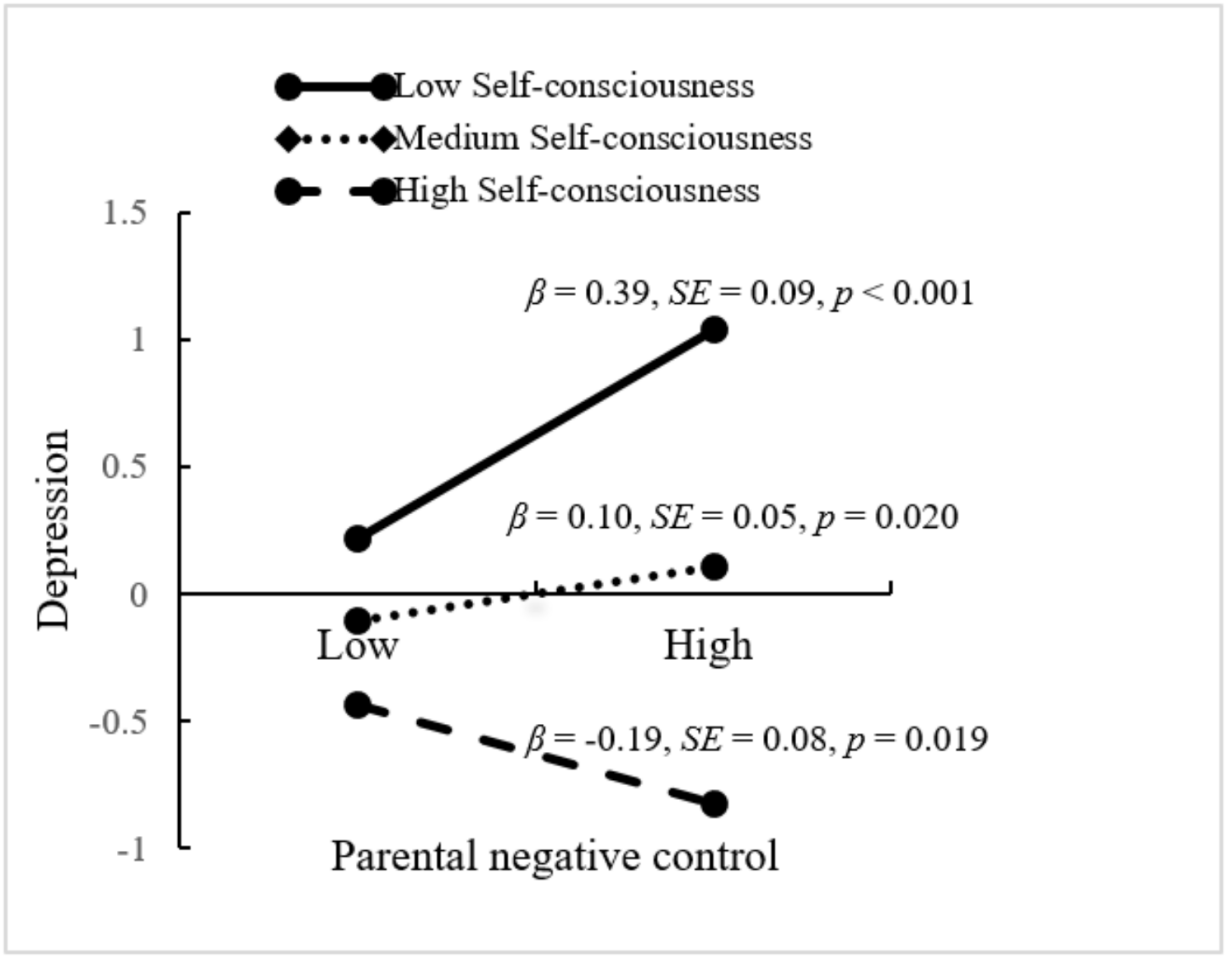
Interactive effect of self-consciousness and parental negative control on depression. Standardized coefficients are reported.

As shown in Table 3, the analysis revealed a significant interaction effect of parental negative control and self-consciousness on depression (*β* = -0.14, *SE* = 0.03, *p* <.001, 95% CI = [-0.201, -0.069]). A follow-up simple slopes test (Fig. 3) indicated that the relationship between parental negative control and depression was stronger when self-consciousness was low (*β* = 0.39, *SE* = 0.09, *p* <.001, 95% CI = [ 0.215, 0.575]) than when self-consciousness was medium (*β* = 0.10, *SE* = 0.05, *p* =.020, 95% CI = [0.016, 0.192]) and high (*β* = -0.19, *SE* = 0.08, *p* =.019, 95% CI = [-0.342, -0.033]). Conditional indirect effect analysis further revealed that the overall indirect effect was significant for adolescents who have low levels of self-consciousness (indirect effect = -0.07, *SE* = 0.02, *p* =.001, 95% CI = [-0.125, -0.034]), medium levels of self-consciousness (indirect effect = -0.02, *SE* = 0.01, *p* =.039, 95% CI = [-0.041, -0.002]), and high levels of self-consciousness (indirect effect = 0.04, *SE* = 0.02, *p* =.036, 95% CI = [0.006, 0.072]).

**Table 3:** The output of the moderated mediation model

	Depression		Perceived school climate (T2)	
	β (SE)	95% CI	β (SE)	95% CI
Main variables				
Parental negative control	0.10* (0.05)	0.016, 0.192	-0.10* (0.05)	-0.184, -0.009
Self-consciousness	-0.50*** (0.05)	-0.608, -0.404		
Parental negative control × Self-consciousness	-0.14*** (0.03)	-0.201, -0.069		
Depression			-0.19*** (0.04)	-0.273, -0.108
Covariates				
Age	-0.23 (0.12)	-0.462, 0.023	-0.21 (0.12)	-0.441, 0.026
Gender	0.17*** (0.04)	0.102, 0.240	0.04 (0.03)	-0.020, 0.108
School type	0.30* (0.12)	0.058, 0.527	0.13 (0.12)	-0.102, 0.363
Socioeconomic status	0.003 (0.04)	-0.065, 0.073	0.09** (0.03)	0.027, 0.147
Perceived school climate (T1)			0.52*** (0.04)	0.437, 0.597
Mediation effect				
Low Self-consciousness	-0.07** (0.02)	-0.125, -0.034		
Medium Self-consciousness	-0.02* (0.01)	-0.041, -0.002		
High Self-consciousness	0.04* (0.02)	0.006, 0.072		

Note: Gender: 0 = male, 1 = female; School type: 0 = junior high school, 1 = senior high school; * *p* < 0.05, ** *p* <.0.01, *** *p* < 0.001; T1 = Time 1, T2 = Time 2

## Discussion

This longitudinal study aims to examine the mediation role of depression in the relationship between parental negative control and adolescent perceived school climate, as well as to evaluate the moderation role of self-consciousness. By exploring these relationships, the study seeks to further the understanding of how individual traits interact with parental behaviors to influence adolescents’ mental health and their perceptions of their school environment. The findings provide adequate support for the two hypotheses proposed.

Consistent with research hypothesis 1, the present study demonstrates that depression mediates the relationship between parental negative control and adolescents’ perceived school climate. Specifically, the findings reveal that adolescents subjected to parental negative control are more likely to exhibit higher levels of depression over time, which aligns with previous research [23, 46]. Factors such as self-criticism, experiential avoidance, rumination, and satisfaction of individuals’ basic psychological needs are identified as the underlying mechanisms through which maladaptive parenting practices heighten adolescents’ internalizing behaviors [10, 71]. The COVID-19 pandemic created an exceptionally stressful environment for families. Under the disruption to life routines, economic instability, and the risk of illness (even mortality), many parents experienced pronounced mental stress, which contributed to increases in maladaptive parenting practices [72, 73]. Concurrently, children and adolescents faced a wide range of challenges, with reduced access to social support, rendering them particularly vulnerable to behavior problems and psychopathologies [74, 75]. Such conditions, which occurred during COVID-19, may intensify the relationship between parental negative control and adolescent depression, resulting in the magnitude of their association being stronger than that in the pre-pandemic context. This calls for future research to examine whether the observed effects persist in post-pandemic conditions to better understand the stability and malleability of parenting influences across different historical contexts.

Moreover, adolescents with higher levels of depression were found to report lower levels of perceived school climate. This is in line with previous studies [42, 43], showcasing the contributing role of depression to negative interpretations of adolescents’ social environment. Depression is often accompanied by cognitive biases. This leads to a tendency to focus on negative stimuli and overlook positive or neutral information in the environment [44]. Hereby, adolescents with depressive symptoms are more likely to fixate on unpleasant experiences at school, such as academic challenges and interpersonal conflicts, construing these events as signs of teachers and peers being unsupportive and unfriendly. Simultaneously, they may internalize these experiences, viewing themselves as lacking worth or competence. In turn, this distorted perception reinforces a negative view of the school environment. By establishing depression as an intermediary mechanism, this study lends support to Bronfenbrenner’s ecological systems theory [20], contributing to the extant body of literature substantiating the salience of microsystem factors, such as parenting, on adolescent development. Furthermore, the depression-induced alteration of perceived emotional connection to school supports the applicability of the cognitive-affective-behavioral model [33] in understanding how adolescents’ emotional state could change their perceptions and engagement within social environments. Although the learning mode for the adolescents in the current study was predominantly in-person, with only a brief one-week period of online learning from T1 to T2, this does not mean COVID-19 had no impact on students’ school experiences. Concerns over virus transmission, undermined sense of security, and uncertainties in general likely impacted students’ emotional connection to the school, including engagement with teachers and peers, through students’ family interaction and personal mental state. While our study does not directly examine these effects, its findings should be interpreted within this broader context.

Supporting research hypothesis 2, the present study demonstrates that self-consciousness moderates the relationship between parental negative control and adolescent depression. Specifically, adolescents with low self-consciousness exhibit heightened vulnerability to the deleterious effects of parental negative control, as evidenced by a stronger positive association with depression. In contrast, adolescents with high self-consciousness demonstrated fewer depressive symptoms, which reveals a potential buffering effect. This finding can be better understood through the lens of self-evaluation processes within the cognitive vulnerability-stress model framework [55]. Adolescents with low self-consciousness typically display a diminished capacity for reflective self-evaluation, potentially leading to a distorted self-concept and heightened susceptibility to external negative influences. These adolescents may be more likely to internalize parental negative control, interpreting it as a reflection of personal inadequacy or failure. This aligns with the hopelessness theory of depression [56] arguing that individuals prone to attribute adverse experiences to stable, internal, and global causes are more vulnerable to developing a sense of hopelessness and depression as they hold the belief that they have no control over the situation. Conversely, adolescents with high levels of self-consciousness demonstrate a greater capacity for adaptive self-evaluation, allowing for cognitive flexibility to reframe parental negative control in a less detrimental light. As such, these adolescents potentially view harmful parental behaviors as situational, temporary, or even constructive, rather than internalizing them as an indication of their value or capabilities. This interpretation aligns with empirical evidence suggesting that positive self-concept serves as a protective factor against depressive symptoms [11, 13].

With the previous studies having investigated self-compassion [13] and self-regulation [11, 12], the current study broadens the scope of self-processes in terms of their moderation effect of parenting on adolescent health outcomes. Supportive of the cognitive vulnerability-stress model [55], the finding suggests that low self-consciousness represents another source of vulnerability that can potentially amplify risks of external stressors on adolescent well-being. In contrast, high self-consciousness represents a source of inner strength, equipping individuals with a greater capacity for adaptive self-evaluation that allows for processing and responding to adversities more positively.

## Implication

As depression emerges as a critical mediator in the relationship between parental negative control and adolescents’ perceptions of their school climate, interventions that aim to improve students’ overall level of connection with the school could consider prioritizing students with depressive symptoms. Mindfulness-based interventions represent a particularly promising avenue, given their demonstrated efficacy in fostering metacognitive awareness and emotional regulation capabilities. For instance, a longitudinal study by Kuyken et al. [76] revealed that structured mindfulness training attenuated depressive symptomatology through enhanced cognitive and emotional flexibility. Furthermore, therapeutic approaches incorporating cognitive-behavioral and family systems perspectives have been proven to effectively ameliorate adolescent depression [77]. By helping mitigate depressive symptoms, students are better able to develop a more balanced and accurate perception of their school.

The finding that self-consciousness serves as a protective psychological mechanism reflects the significance of fostering this intrapersonal attribute. Structured programs designed to cultivate adaptive and functional self-processes, such as self-regulation, self-compassion, and self-control, could incorporate the component of self-consciousness that aims to enhance self-reflective capacities, promote positive self-evaluation, and teach adaptive cognitive reframing. Adolescents, particularly those with lower levels of self-consciousness would benefit from such interventions and develop a flexible interpretation of and productive responses to external stressors, reducing their vulnerability to psychopathological symptoms.

The study also highlights the need for parent-focused interventions aimed at reducing negative parenting practices given their harmful impacts on adolescents. Approaches could involve awareness cultivation that helps parents recognize the potential consequences of overly controlling or intrusive behaviors and adopt more nurturing and autonomy-promoting parenting behaviors. Behavioral skill training can equip parents with techniques geared toward effective communication and conflict resolution and enable them to engage in constructive parent-adolescent interactions. Interventions can also benefit from focusing on improving interpersonal dynamics by enhancing parental empathy and responsiveness as an emotionally supportive home environment empowers adolescents to positively face stressors that otherwise could contribute to mental and behavioral issues.

## Limitation

We must acknowledge several limitations in this study. First, while this study employed a longitudinal design to examine the effects of parental negative control and depression on perceived school climate, it did not fully account for the bidirectional relationship between depression and perceived school climate [36, 38]. Notably, both depression and perceived school climate were measured at the same time point (T2), which limits the ability to establish a predictive relationship or draw causal conclusions about the directionality between these variables. Future research should adopt a multi-wave longitudinal design with measurements of variables at multiple time points to explore the cross-lagged relationships among parental negative control, depression, and perceived school climate. Such approaches would provide greater clarity on the temporal dynamics between these constructs. Second, this study relied solely on adolescents’ self-reports for data. Adolescents’ perceptions may not accurately reflect reality, particularly among those with depressive symptoms, as cognitive biases associated with depression can distort evaluations of others and external contexts [44]. While self-reports provide valuable insights into adolescents’ subjective experiences, the reliance on a single source of data introduces the risk of social desirability biases. To minimize such limitations, future studies should incorporate multiple sources of information, such as parent-reported data on parenting behaviors, teacher assessments of school climate, and objective measures, such as physiological indicators of adolescent depression. The results indeed reveal a negative association between depressive symptoms and perceived school climate, consistent with the assumption. However, an alternative interpretation warrants consideration, that is, adolescents’ perceptions of school climate may accurately reflect their lived experiences rather than being distorted by depressive symptoms. This possibility introduces a limitation that the study cannot determine whether perceptions are biased or represent reality. Future research could employ multi-informant designs, such as teacher and peer reports and observational data, and incorporate qualitative methods including interviews or focus groups, to gain a more accurate picture regarding whether adolescents’ perceptions align with actual school conditions.

## Conclusions

This study contributes to the literature by examining a moderated mediation model that sheds light on the interplay between parental negative control, depression, self-consciousness, and perceived school climate in adolescents. The findings demonstrate that depression mediates the link between parental negative control and perceived school climate, while self-consciousness serves as a protective factor, moderating the relationship between parental negative control and depression. Given the global prevalence of mental health problems among the young population and the growing amount of time they spend in school as they age, it is imperative to preemptively intervene with families involved in harmful parenting practices, adolescents experiencing psychopathological symptoms, and those with low self-consciousness, by a strategic approach to enhance family dynamics and individuals’ functioning of self-processes.

## Data Availability

The datasets generated during and analyzed during the current study are not publicly available due to the containing information that could compromise research participant privacy and consent but are available from the corresponding author upon reasonable request.

## Footnotes

1 Mplus does not generate commonly used fit indices such as χ², CFI, TLI, RMSEA, and SRMR for latent moderated mediation models.

## References

[1] Verner-Filion J, Véronneau M, Vaillancourt M, Mathys C. Perceived school climate and school grades in secondary school students: the mediating effect of self-determined motivation. Contemp Educ Psychol. 2023;74:102202. 10.1016/j.cedpsych.2023.102202.

[2] Zysberg L, Schwabsky N. School climate, academic self-efficacy and student achievement. Educ Psychol-Uk. 2020;41(4):467–82. 10.1080/01443410.2020.1813690.

[3] Hoferichter F, Hirvonen R, Kiuru N. The development of school well-being in secondary school: high academic buoyancy and supportive class- and school climate as buffers. Learn Instr. 2021;71:101377. 10.1016/j.learninstruc.2020.101377.

[4] Thapa A, Cohen J, Guffey S, Higgins-D’Alessandro A. A review of school climate research. Rev Educ Res. 2013;83(3):357–85. 10.3102/0034654313483907.

[5] Aldridge JM, McChesney K. (2018). The relationships between school climate and adolescent mental health and wellbeing: a systematic literature review. Int J Educ Res. 2018;88:121–45. 10.1016/j.ijer.2018.01.012

[6] Casey BJ, Jones RM, Hare TA. The adolescent brain. Ann N Y Acad Sci. 2008;1124:111–26. 10.1196/annals.1440.010. 18400927 10.1196/annals.1440.010 PMC2475802

[7] Steinberg L. A dual systems model of adolescent risk-taking. Dev Psychobiol. 2010;52(3):216–24. 10.1002/dev.20445. 20213754 10.1002/dev.20445

[8] Tariq A, Quayle E, Lawrie SM, Reid C, Chan SWY. Relationship between early maladaptive schemas and anxiety in adolescence and young adulthood: a systematic review and meta-analysis. J Affect Disord. 2021;295:1462–73. 10.1016/j.jad.2021.09.031. 34563389 10.1016/j.jad.2021.09.031

[9] Lansford JE, Laird RD, Pettit GS, Bates JE, Dodge KA. Mothers’ and fathers’ autonomy-relevant parenting: longitudinal links with adolescents’ externalizing and internalizing behavior. J Youth Adolesc. 2014;43(11):1877–89. 10.1007/s10964-013-0079-2. 24337705 10.1007/s10964-013-0079-2 PMC4061285

[10] Xiao H, Wu Y, Chen Z, Nie Y. Which need satisfaction better explains the relation between parental psychological control and adolescent behavior problems: a parallel multi-mediator model based on general strain theory. Child Youth Serv Rev. 2024;163:107779. 10.1016/j.childyouth.2024.107779.

[11] Crespo LM, Trentacosta CJ, Udo-Inyang I, Northerner L, Chaudhry K, Williams A. Self-regulation mitigates the association between household chaos and children’s behavior problems. J Appl Dev Psychol. 2019;60:56–64. 10.1016/j.appdev.2018.10.005. 31772417 10.1016/j.appdev.2018.10.005 PMC6879109

[12] Way N, Reddy R, Rhodes J. Students’ perceptions of school climate during the middle school years: associations with trajectories of psychological and behavioral adjustment. Am J Community Psychol. 2007;40(3–4):194–213. 10.1007/s10464-007-9143-y. 17968655 10.1007/s10464-007-9143-y

[13] Liu Q, Hu Y. (2020). Self-compassion mediates and moderates the association between harsh parenting and depressive symptoms in Chinese adolescent. Curr Psychol. 2020;42(19):16036–48. 10.1007/s12144-020-01034-2

[14] Nie YG, Li JB, Dou K, Situ QM. The associations between self-consciousness and internalizing/externalizing problems among Chinese adolescents. J Adolesc. 2014;37(5):505–14. 10.1016/j.adolescence.2014.04.002. 24931553 10.1016/j.adolescence.2014.04.002

[15] Zhao M, Ford T, Panayiotou M, Karl A. Developmental pathways of depressive symptoms via parenting, self-evaluation and peer relationships in young people from 3 to 17 years old: evidence from ALSPAC. Soc Psychiatry Psychiatr Epidemiol. 2023;58(6):907–17. 10.1007/s00127-022-02416-6. 36708401 10.1007/s00127-022-02416-6 PMC10241697

[16] Kammeyer-Mueller JD, Judge TA, Scott BA. The role of core self-evaluations in the coping process. J Appl Psychol. 2009;94(1):177–95. 10.1037/a0013214. 19186903 10.1037/a0013214

[17] Sun Z, Ding W, Chu X, et al. The relationship between perceived childhood harsh parental discipline and cyberbullying among college students: a moderated mediation model. J Adult Dev. 2022;30(4):321–33. 10.1007/s10804-022-09432-5.

[18] Liu F, Wang N, Chui H, Wang X, Chen N. (2023). The association between left-behind children status and peer victimization: self-esteem and perceived social support as potential moderators. J Aggress Maltreat T. 2023;33(3):351–68. 10.1080/10926771.2023.2222663

[19] Luo H, Yang Q, Chen J, Li Z, Nie Y. The relationship between social exclusion and impulsive buying of college students: the mediating role of celebrity worship and the moderating role of self-control. J Psychol Sci. 2022;45(3):657–64. https://jps.ecnu.edu.cn/CN/Y2022/V45/I3/657.

[20] Bronfenbrenner U. The ecology of human development: experiments by nature and design. Cambridge, MA: Harvard University Press; 1979.

[21] Lin W, Liang H, Jiang H, Mohd Nasir MA, Zhou H. Why is smartphone addiction more common in adolescents with harsh parenting? Depression and experiential Avoidance’s multiple mediating roles. Psychol Res Behav Manag. 2023;16:4817–28. 10.2147/PRBM.S428167. 38047152 10.2147/PRBM.S428167 PMC10693199

[22] Wang D, Zhou M, Hu Y. The relationship between harsh parenting and smartphone addiction among adolescents: serial mediating role of depression and social pain. Psychol Res Behav Manag. 2024;17:735–52. 10.2147/PRBM.S438014. 38410380 10.2147/PRBM.S438014 PMC10896639

[23] Tang A, Deng X, Du X, Wang M. (2018). Harsh parenting and adolescent depression: mediation by negative self-cognition and moderation by peer acceptance. School Psychol Int. 2018;39(1):22–37. 10.1177/0143034317709066.

[24] Jin C, Zou H. The relationship between parental monitoring and adolescents’ online deviant behavior: personality as a mediator. Chin J Special Educ. 2013;6:63–8.

[25] Zhang W, Zou H, Li X. Current status of adolescents parental monitoring and its effect on their social adjustment. Psychol Dev Educ. 2011;27(3):267–73. https://devpsy.bnu.edu.cn/CN/Y2011/V27/I3/267.

[26] Geng J, Wang X, Wang Y, Lei L, Wang P. If you love me, you must do… Parental psychological control and cyberbullying perpetration among Chinese adolescents. J Interpers Violence.2022;37(9–10):NP7932–NP7957. 10.1177/0886260520978185. 10.1177/0886260520978185 33213265

[27] Wang Q, Pomerantz EM, Chen H. The role of parents’ control in early adolescents’ psychological functioning: a longitudinal investigation in the united States and China. Child Dev. 2007;78(5):1592–610. 17883450 10.1111/j.1467-8624.2007.01085.x

[28] Ha JH, Jue J. The mediating effect of emotion Inhibition and emotion regulation between adolescents’ perceived parental psychological control and depression. Sage Open. 2018;8(3). 10.1177/2158244018793680.

[29] Yu X, Fu X, Yang Z, et al. Bidirectional relationship between parental psychological control and adolescent maladjustment. J Adolesc. 2021;92:75–85. 10.1016/j.adolescence.2021.08.007. 34433117 10.1016/j.adolescence.2021.08.007

[30] Soenens B, Park SY, Vansteenkiste M, Mouratidis A. Perceived parental psychological control and adolescent depressive experiences: a cross-cultural study with Belgian and South-Korean adolescents. J Adolesc. 2012;35(2):261–72. 10.1016/j.adolescence.2011.05.001. 21620464 10.1016/j.adolescence.2011.05.001

[31] Beck AT. Depression: clinical, experimental, and theoretical aspects. Philadelphia, PA: University of Pennsylvania; 1967.

[32] Beck AT. The evolution of the cognitive model of depression and its Neurobiological correlates. Am J Psychiatry. 2008;165(8):969–77. 10.1176/appi.ajp.2008.08050721. 18628348 10.1176/appi.ajp.2008.08050721

[33] Pachankis JE. The psychological implications of concealing a stigma: a cognitive-affective-behavioral model. Psychol Bull. 2007;133(2):328–45. 10.1037/0033-2909.133.2.328. 17338603 10.1037/0033-2909.133.2.328

[34] Wang M, Degol JL. School climate: a review of the construct, measurement, and impact on student outcomes. Educ Psychol Rev. 2016;28(2):315–52. 10.1007/s10648-015-9319-1.

[35] Cohen J, Mccabe EM, Michelli NM, Pickeral T. School climate: research, policy, practice, and teacher education. Teach Coll Rec. 2009;111(1):180–213. 10.1177/016146810911100108.

[36] Nie Q, Yang C, Teng Z, et al. Longitudinal association between school climate and depressive symptoms: the mediating role of psychological Suzhi. Sch Psychol. 2020;35(4):267–76. 10.1037/spq0000374. 32673054 10.1037/spq0000374

[37] Gotlib IH, Joormann J. Cognition and depression: current status and future directions. Annu Rev Clin Psychol. 2010;6:285–312. 10.1146/annurev.clinpsy.121208.131305. 20192795 10.1146/annurev.clinpsy.121208.131305 PMC2845726

[38] Ding D, Pan M, Tang Q, Zhang J. Longitudinal association between school climate and psychological flexibility and mental health: a random intercept cross lagged panel model. J Psychopathol Behav. 2024;46(4):916–24. 10.1007/s10862-024-10151-2.

[39] Zhai B, Li D, Li X, et al. Perceived school climate and problematic internet use among adolescents: mediating roles of school belonging and depressive symptoms. Addict Behav. 2020;110:106501. 10.1016/j.addbeh.2020.106501. 32634681 10.1016/j.addbeh.2020.106501

[40] Fabbri C, Powell-Jackson T, Leurent B, et al. School violence, depression symptoms, and school climate: a cross-sectional study of Congolese and Burundian refugee children. Confl Health. 2022;16(1):42. 10.1186/s13031-022-00475-9. 35870935 10.1186/s13031-022-00475-9 PMC9308201

[41] Jia Y, Way N, Ling G, et al. The influence of student perceptions of school climate on socioemotional and academic adjustment: a comparison of Chinese and American adolescents. Child Dev. 2009;80(5):1514–30. 10.1111/j.1467-8624.2009.01348.x. 19765015 10.1111/j.1467-8624.2009.01348.x

[42] Platt B, Cohen Kadosh K, Lau JY. The role of peer rejection in adolescent depression. Depress Anxiety. 2013;30(9):809–21. 10.1002/da.22120. 23596129 10.1002/da.22120

[43] Quiroga CV, Janosz M, Bisset S, Morin AJS. Early adolescent depression symptoms and school dropout: mediating processes involving self-reported academic competence and achievement. J Educ Psychol. 2013;105(2):552–60. 10.1037/a0031524.

[44] Rudolph KD, Clark AG. Conceptions of relationships in children with depressive and aggressive symptoms: social-cognitive distortion or reality? J Abnorm Child Psychol. 2001;29(1):41–56. 10.1023/a:1005299429060. 11316334 10.1023/a:1005299429060

[45] Zhu Y, Wang Q, Liu J, Huang J. Parental psychological control and depression, anxiety among adolescents: the mediating role of bedtime procrastination and moderating role of neuroticism. Arch Psychiatr Nurs. 2024;51:1–9. 10.1016/j.apnu.2024.05.002. 39034062 10.1016/j.apnu.2024.05.002

[46] Wang M, Chen Q, Deng X. (2024). Longitudinal pathways from harsh parenting to adolescent depression via internal working models: the moderating role of adolescent trait mindfulness. J Soc Pers Relat. 2024;41(10):3109–22. 10.1177/02654075241263873

[47] Quach AS, Epstein NB, Riley PJ, Falconier MK, Fang X. Effects of parental warmth and academic pressure on anxiety and depression symptoms in Chinese adolescents. J Child Fam Stud. 2015;24(1):106–16. 10.1007/s10826-013-9818-y.

[48] Wang Z, Jiang S. Influence of parental neglect on cyberbullying perpetration: moderated mediation model of smartphone addiction and self-regulation. Health Soc Care Community. 2022;30(6):2372–82. 10.1111/hsc.13787. 35298055 10.1111/hsc.13787

[49] Morange-Majoux F, Dellatolas G, Hervé PY. Exploring self-consciousness from self- and other-image recognition in the mirror: concepts and evaluation. Front Psychol. 2019;10:719. 10.3389/fpsyg.2019.00719. 31133909 10.3389/fpsyg.2019.00719 PMC6524719

[50] Rochat P. Five levels of self-awareness as they unfold early in life. Conscious Cogn. 2003;12(4):717–31. 10.1016/S1053-8100(03)00081-3. 14656513 10.1016/s1053-8100(03)00081-3

[51] Delvecchio E, Mabilia D, Lis A, Mazzeschi C, Nie Y, Li JB. From China to Italy: validation of the adolescent self-consciousness questionnaire. Eur J Dev Psychol. 2013;11(1):120–28. 10.1080/17405629.2013.831760.

[52] Baumeister RF, Vohs KD. Handbook of self-regulation: research, theory, and applications. New York, NY: Guilford Press; 2004.

[53] Nie Y, Li J, Vazsonyi AT. Self-control mediates the associations between parental attachment and prosocial behavior among Chinese adolescents. Pers Indiv Differ. 2016;96:36–9. 10.1016/j.paid.2016.02.077.

[54] Nie YG. Exploration of adolescents’ self-consciousness. Beijing: China Social Science; 2016.

[55] Hankin BL. Cognitive vulnerability-stress model of depression during adolescence: investigating depressive symptom specificity in a multi-wave prospective study. J Abnorm Child Psychol. 2008;36(7):999–1014. 10.1007/s10802-008-9228-6. 18437551 10.1007/s10802-008-9228-6 PMC2763423

[56] Abramson LY, Metalsky GI, Alloy LB. Hopelessness depression: a theory-based subtype of depression. Psychol Rev. 1989;96(2):358–72. 10.1037/0033-295X.96.2.358.

[57] Abramson LY, Alloy LB, Hankin BL, Haeffel GJ, MacCoon DG, Gibb BE. Cognitive vulnerability-stress models of depression in a self-regulatory and Psychobiological context. In: Gotlib IH, Hammen CL, editors. Handbook of depression. New York, NY: The Guilford Press; 2002. pp. 268–94.

[58] Gibb BE, Coles ME. Cognitive vulnerability-stress models of psychopathology: a developmental perspective. In: Hankin BL, Abela JRZ, editors. Development of psychopathology: a vulnerability-stress perspective. Thousand Oaks, CA: Sage Publications, Inc; 2005. pp. 104–35.

[59] Barreto IS, Teodoro MLM, Ohno PM, Froeseler MVG. Cognitive vulnerability and stress for emotional and behavioral problems in children and adolescents: a longitudinal study. J Cogn Psychother. 2018;32(4):272–84. 10.1891/0889-8391.32.4.272. 32746407 10.1891/0889-8391.32.4.272

[60] Hamilton JL, Stange JP, Abramson LY, Alloy LB. Stress and the development of cognitive vulnerabilities to depression explain sex differences in depressive symptoms during adolescence. Clin Psychol Sci. 2015;3(5):702–14. 10.1177/2167702614545479. 26509106 10.1177/2167702614545479 PMC4617303

[61] Jankowski KF, Batres J, Scott H, Smyda G, Pfeifer JH, Quevedo K. Feeling left out: depressed adolescents May atypically recruit emotional salience and regulation networks during social exclusion. Soc Cogn Affect Neurosci. 2018;13(8):863–76. 10.1093/scan/nsy055. 30059994 10.1093/scan/nsy055 PMC6123522

[62] Andresen EM, Malmgren JA, Carter WB, Patrick DL. Screening for depression in well older adults: evaluation of a short form of the CES-D (Center for epidemiologic studies depression Scale). Am J Prev Med. 1994;10(2):77–84. 8037935

[63] Nie YG, Ding L. The characters of adolescent self-consciousness and its relationship with social adaptive behavior. Psychol Dev Educ. 2009;25(2):47–54. https://devpsy.bnu.edu.cn/CN/Y2009/V25/I2/47.

[64] Bear GG, Yang C, Mantz L, Pasipanodya E, Hearn S, Boyer D. Technical manual for Delaware school survey: scales of school climate, bullying victimization, student engagement, and positive, punitive, and social emotional learning techniques. Delaware positive behavior support (DE-PBS) and school climate transformation projects. Center for Disabilities Studies; 2014.

[65] Welsh RO, Rodriguez LA, Joseph B. Examining student perceptions of school climate, school personnel, and school discipline: evidence from new York City. J Sch Psychol. 2024;107:101361. 10.1016/j.jsp.2024.101361. 39645320 10.1016/j.jsp.2024.101361

[66] Hoyle RH. Handbook of structural equation modeling. New York, NY: Guilford Press; 2012.

[67] Kline RB. Principles and practice of structural equation modeling. 4th ed. New York, NY: Guilford Press; 2015.

[68] Hair JF Jr, Black WC, Babin BJ, Anderson RE. Multivariate Data Analysis. 7th ed. Pearson; 2010.

[69] Podsakoff PM, MacKenzie SB, Lee JY, Podsakoff NP. Common method biases in behavioral research: a critical review of the literature and recommended remedies. J Appl Psychol. 2003;88(5):879–903. 10.1037/0021-9010.88.5.879. 14516251 10.1037/0021-9010.88.5.879

[70] Maslowsky J, Jager J, Hemken D. Estimating and interpreting latent variable interactions: A tutorial for applying the latent moderated structural equations method. Int J Behav Dev. 2015;39(1):87–96. 10.1177/0165025414552301. 26478643 10.1177/0165025414552301 PMC4606468

[71] Bleys D, Soenens B, Claes S, Vliegen N, Luyten P. Parental psychological control, adolescent self-criticism, and adolescent depressive symptoms: A latent change modeling approach in Belgian adolescents. J Clin Psychol. 2018;74(10):1833–53. 10.1002/jclp.22632. 29696640 10.1002/jclp.22632

[72] Kerr ML, Rasmussen HF, Fanning KA, Braaten SM. Parenting during COVID-19: A study of parents’ experiences across gender and income levels. Fam Relat. 2021;70(5):1327–42. 10.1111/fare.12571. 34548726 10.1111/fare.12571 PMC8444790

[73] Xiao H, Mao J, Chen J, Wei J, He J, Nie Y. The mediation roles of self-regulation and problematic internet use: how maladaptive parenting during the COVID‐19 pandemic influenced adolescents’ academic procrastination in the postpandemic era. J Adolesc. 2024;96(7):1458–72. 10.1002/jad.12355. 38812273 10.1002/jad.12355

[74] Breaux R, Cash AR, Lewis J, Garcia KM, Dvorsky MR, Becker SP. Impacts of COVID-19 quarantine and isolation on adolescent social functioning. Curr Opin Psychol. 2023;52:101613. 10.1016/j.copsyc.2023.101613. 37364468 10.1016/j.copsyc.2023.101613 PMC10232930

[75] Loades ME, Chatburn E, Higson-Sweeney N, et al. Rapid systematic review: the impact of social isolation and loneliness on the mental health of children and adolescents in the context of COVID-19. J Am Acad Child Adolesc Psychiatry. 2020;59(11):1218–39. 10.1016/j.jaac.2020.05.009. 32504808 10.1016/j.jaac.2020.05.009 PMC7267797

[76] Kuyken W, Weare K, Ukoumunne OC, et al. Effectiveness of the mindfulness in schools programme: non-randomised controlled feasibility study. Br J Psychiatry. 2013;203(2):126–31. 10.1192/bjp.bp.113.126649. 23787061 10.1192/bjp.bp.113.126649

[77] Carr A. Family therapy for adolescents: A research-informed perspective. Aust Nz J Fam Ther. 2016;37(4):467–79. 10.1002/anzf.1184.

